# Classification of chronic cough by systematic treatment cascade trial starting with beta agonist

**DOI:** 10.1186/1745-9974-9-4

**Published:** 2013-02-07

**Authors:** Hideyasu Shimizu, Masamichi Hayashi, Yuji Saito, Yuki Mieno, Yasuo Takeuchi, Fumihiko Sasaki, Hiroki Sakakibara, Kensei Naito, Mitsushi Okazawa

**Affiliations:** 1 Department of Internal Medicine, Division of Respiratory Medicine and Clinical Allergy, Fujita Health University, 1-98 Dengakugakubo Kutsukakecho, Toyoake, 470-1192, Japan; 2 Department of Otolaryngology, Fujita Health University, Toyoake, 470-1192, Japan

**Keywords:** Airway hyperresponsiveness, β_2_ agonist, Bronchial asthma, Cough variant asthma, Non-hyperresponsive and β_2_ agonist responsive cough, Gastroesophageal reflex disease, Sinobronchial syndrome, Atopic cough, Postnasal drip, Chronic cough

## Abstract

**Background:**

Chronic cough is one of the most challenging symptoms to diagnose and treat, not only because of the variety of underlying disorders but also its varying susceptibility to treatments. Etiological studies of chronic cough vary depending on the clinical settings and the particular interests of investigators.

**Objectives:**

The purposes of this study were first to categorize the etiology of chronic cough by its response to systematic diagnostic treatments starting from the β_2_ agonist and second to sub-categorize β_2_ agonist responsive cough (BRC) by the airway hyperresponsiveness.

**Methods:**

One hundred and eighty-four never-smokers received the maximal dose of procaterol to diagnose BRC. BRC was sub-categorized into two groups with or without airway hyperresponsiveness measured by the methacholine challenge test. Sinobronchial syndrome (SBS) was diagnosed by postnasal drip symptoms and by the response to clarythromycin and carbocysteine. Atopic cough (AC) was diagnosed by the evidence of atopy and the response to cetirizine hydrochloride. Gastroesophageal reflux disease (GERD) was diagnosed by the response to rabeprazole sodium. Since we did not investigate eosinophil counts in the tissue or in the induced sputum, no diagnosis of eosinophilic bronchitis was made.

**Results:**

One hundred and nine patients had BRC. Twenty-three of them had bronchial asthma (BA), 53 had cough variant asthma (CVA) and 33 had non-hyperresponsive BRC (NHBRC). Thirty-one patients had GERD, 27 had AC and 14 had SBS. Twenty-five patients had more than one diagnosis in combination, while 6 had other miscellaneous diseases. Twelve patients were undiagnosed and 11 dropped out of the study.

**Conclusions:**

The majority of chronic cough was BRC. NHBRC was a new chronic cough entity. GERD is a common cause of chronic cough in Japan, as in Western countries. AC and SBS are also causes of chronic cough in Japan.

**Trial registration:**

University hospital medical information network (UMIN 000007483)

## Background

Although cough is the most effective defense mechanism for eliminating foreign materials, including numerous pathogens from the airways, it deteriorates health-related quality of life [1]. Therefore, cough is the most common symptom for which patients seek medical attention [2].

Chung and Pavord classified the chronic cough into corticosteroid-responsive eosinophilic airway diseases, such as asthma (BA), cough variant asthma (CVA), eosinophilic bronchitis (EB) and corticosteroid resistant disorders such as gastro-esophageal reflux disease (GERD), and the postnasal drip syndrome (PNDS) or rhino-sinusitis [3]. Although all the eosinophilic airway diseases respond to corticosteroid treatment, it is not clear whether these disorders are separate entities, or all one disorder with different levels of severity. Indeed, ~ 30% of CVA is reported to develop into BA [4,5], suggesting that some types of CVA could be a precursor of BA. On the other hand, some types of PNDS, such as allergic rhinitis and AC, also respond to corticosteroid [6]. Obviously, the prognosis and impact on health-related quality of life from these conditions are quite different from bronchial asthma, and it is not easy to decide how long corticosteroid treatment should be continued. Therefore, investigating how to differentiate corticosteroid-responsive cough from other types of cough is very worthwhile.

Etiological studies of chronic cough vary depending on the clinical setting, the particular interest, the age of patients, and the local definition of diseases [4,7-10]. For example, sinobronchial syndrome (SBS) is more common in Japan, whereas GERD is less common [11].

The purposes of this study were to categorize the etiology of chronic cough by its response to the systematic diagnostic treatments starting from a high dose of an inhaled β_2_ agonist, and secondarily to sub-categorize the β_2_ agonist responsive cough (BRC) by airway hyperresponsiveness using the methacholine challenge test.

## Methods

**Figure 1:**
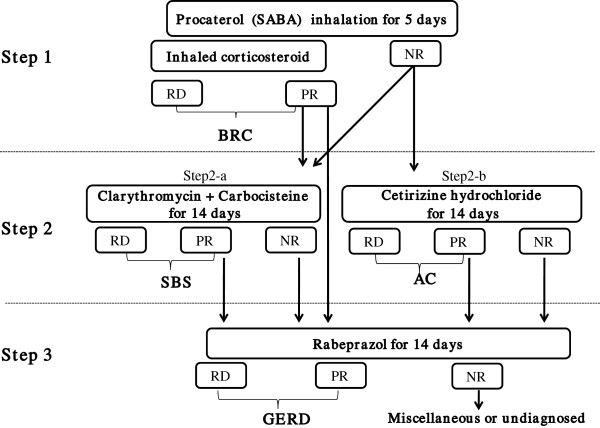
**Diagnostic treatment cascade.** RD, PR and NR stand for Responder, Partial-responder and Non-responder, respectively. BRC, AC, SBS and GERD stand for β_2_ agonist responsive cough, Atopic cough, Sinobronchial syndrome and Gastroesophageal reflux disease, respectively.

This study was approved by the ethics committee of Fujita Health University, and written informed consent was obtained from all the patients who participated. One hundred eighty-four consecutive never-smokers with no chest radiograph abnormalities, who visited the cough clinic of Fujita Health University complaining of chronic cough lasting for more than 8 weeks, were enrolled. Our cough clinic is specialized for patients whose major complaint is chronic cough. More than 90% of the enrolled patients visited our clinic without referrals from other doctors. Forty-seven were male and 137 were female. The mean age of males and females was 43 ± 14 years and 45 ± 16 years, respectively. When a patient was taking angiotensin converting enzyme inhibitor (ACEI) for the treatment of hypertension, the medication was changed. After obtaining a medical history, laboratory examinations including serum IgE levels and methacholine challenge test were performed. The methacholine challenge test was performed according to the guideline [12]. Briefly, the forced expiratory volume in one second (FEV_1_) was measured after 2 minutes’ inhalation of saline and then the doubling dose of methacholine was started from 0.031 mg/ml. If the final doubling dose of 16 mg/ml was reached, a higher dose of 20 mg/ml was added in this study. The methacholine challenge test was diagnosed to be positive, when FEV_1_ decreased by more than 20% after inhalation of an increasing concentration of methacholine (PC_20_) of 8 mg/ml or less. Then, the stepwise diagnostic treatments were applied (Figure 1). In each step, the patient was subjectively classified as a responder (RD) when the cough completely diminished. The patient was classified as a partial-responder (PR) when the cough had clearly decreased, yet persisted even with the treatment. The patient was classified as a non-responder (NR) when the cough did not decrease at all by the treatment. In step 1, all the patients received a metered-dose short-acting β_2_ agonist inhalation to start with. They received two puffs of procaterol (20 μg), 4 times per day for 5 days using an procaterol inhaler (Meptin Air Inhaler^R^, Otsuka Pharmaceutical Co. Ltd., Japan). This is the maximal daily dose for Japanese, and all the patients were compliant with the protocol, although some experienced tremor or palpitation. Patients who responded to procaterol by the end of the 5^th^ day underwent an airway reversibility test 24 hours after cessation of the procaterol inhaler. The reversibility test was performed according to the guideline [13]. Briefly, forced expiratory maneuvers were repeated before and 15 minutes after nebulization of salbutamol solution (0.65 mg/ml) for 5 minutes. The reversibility test was considered positive when FEV_1_ increased by more than 12% and 200 ml from the baseline values. Thereafter, patients who responded to procaterol received an inhaled corticosteroid (ICS), either fluticasone propionate (400 μg), budesonide (800 μg) or beclomethasone dipropionate (400 μg) throughout the protocol. The final assessment of step 1 was made 2 weeks after inhalation of ICS. PRs at step 1 who had postnasal drip (PND) and phlegm received step 2-a treatment of oral administration of 400 mg clarithromycin and 1500 mg carbocysteine per day for 14 days in addition to ICS. PRs at step2-a received step 3 treatment of oral administration of 20 mg rabeprazole sodium per day for 14 days on top of ICS and step 2-a treatment. PRs at step1 who did not have PND and phlegm skipped both step 2 treatments and received step 3 treatment of oral administration of 20 mg rabeprazole sodium per day on top of ICS. For NRs at step 1 who had a previous history of allergic diseases, increased serum total IgE levels (>130 IU/ml) or specific IgE antibodies measured by ELISA method (MAST26, Hitachi Chemical Co. Ltd., Tokyo), ICS was not used, and the step 2-b treatment of oral administration of 10 mg cetirizine hydrochloride per day for 14 days was applied. NRs at step 1 who had PND and phlegm received step 2-a treatments. NRs both to step 2-a and 2-b treatments proceeded to step 3 treatment in which 20 mg of rabeprazole sodium alone was used per day for 14 days.

### Diagnostic criteria

RDs and PRs at step 1 were diagnosed with a β_2_ agonist responsive cough (BRC). BRC patients who had PC_20_ of 8 mg/ml or less were classified as having hyperresponsive and β_2_ agonist responsive cough (HBRC). Among HBRC, patients with a previous history of bronchial asthma or asthmatic symptoms such as episodic wheeze or dyspnea were categorized with bronchial asthma (BA). Patients who had a positive methacholine challenge test without any asthmatic symptoms such as wheezing or dyspnea were diagnosed with cough variant asthma (CVA). Patients without any asthmatic symptoms who failed to undergo the methacholine challenge test were also categorized as CVA. Patients were diagnosed to have non-hyperresponsive BRC (NHBRC) when they responded to the β_2_ agonist but their PC_20_ was 20 mg/ml or over. Patients with postnasal drip (PND) and phlegm were diagnosed with sinobronchial syndrome (SBS) [9] when they responded to the oral administration of clarithromycin and carbocysteine. Patients were diagnosed as atopic cough (AC) [9] when they had either a high level of serum IgE (>130 IU/ml), or specific IgE antibodies measured by the ELISA method, or other allergic disorders such as allergic rhinitis or food allergy, and responded to oral administration of cetirizine hydrochloride but not to inhaled β_2_ agonist. Patients were diagnosed with gastroesophageal reflux disease (GERD) when they responded to the oral administration of rabeprazole sodium. NRs to all the treatments at step 1–3 received further examinations such as computed tomography, bronchoscopy, sputum bacteriologies, or serological examinations.

### Statistics

RDs in step 1–3 were selected for a basic comparison. The duration of cough, serum IgE level, a symptom of PND, PC_20_, %VC, and % FEV_1_/FVC ratio, and flow volume parameters were compared using ANOVA and post hoc test with Bonferroni correction. Changes in the flow volume parameters of BRCs before and after the airway reversibility test were compared using paired *t*-test. The magnitude of change in flow volume parameters were compared using ANOVA and post hoc test with Bonferroni correction. A p value less than 0.05 was considered to be significant. All the statistics were performed using StatView5.0 (HULINKS Inc. Tokyo).

## Results

### Classification of chronic cough

Eleven patients dropped out of the analysis because they did not complete the protocol. One-hundred and nine patients were with BRC (Table 1), and eighty-six of them received methacholine challenge tests. The others did not undergo the tests because of increasing cough during a test or their refusal to take the examination. No patients showed worsening of cough after switching from SABA to ICS in step 1 procedure. Twenty-three patients had a previous history of bronchial asthma, mostly during childhood, or had asthmatic symptoms such as wheezing and were diagnosed with BA. Nineteen of them experienced complete diminution of chronic cough after step 1 treatment but the other 4 patients required step 2 or 3 treatments in addition to step 1 treatment. They were diagnosed with BA + SBS or BA + GERD. Fifty-three patients were diagnosed with CVA. Forty-seven of them experienced complete diminution of chronic cough after step 1 treatment but the other 6 patients were diagnosed either with CVA + SBS or with CVA + GERD. Thirty-three patients were diagnosed with NHBRC. Thirty of them experienced complete diminution by step 1 treatment, while the other three were diagnosed either with NHBRC + SBS or NHBRC + GERD.

**Table 1:** Classification of BRC

		**BRC 109 (21/88)**			
**BA**	**23 (6/17)**	**CVA**	**53 (8/45)**	**NHBRC**	**33 (7/26)**
BA	19 (5/14)	CVA	47 (8/39)	NHBRC	30 (7/23)
BA + SBS	2 (0/2)	CVA + SBS	2 (0/2)	NHBRC + SBS	2 (0/2)
BA + GERD	2 (1/1)	CVA + GERD	4 (0/4)	NHBRC + GERD	1 (0/1)

*BA*, Bronchial asthma; *CVA*, Cough variant asthma; *NHBRC*, Non-hyperresponsive and β_2_ agonist responsive cough; numbers within parentheses indicate males and females.

Sixty-four patients were diagnosed with non-BRC (Table 2). Among them, thirty-three received methacholine challenge tests. The others did not undergo the tests because of increasing cough during a test or their refusal to take the examination. Twenty-seven of 64 patients responded to the treatment of step 2-b and were diagnosed with AC. The cough completely diminished in 17 of them. Others were diagnosed either with AC + SBS + GERD or AC + GERD. Seven patients were diagnosed with SBS because they responded to the treatment of step 2-a. Two of them required step 3 treatment before their cough diminished and were diagnosed with SBS + GERD. Twelve patients were diagnosed with GERD because their cough completely diminished only by step 3 treatment. Six patients were diagnosed as Miscellaneous (one ACEI-induced cough, 2 non-tuberculous mycobacterial infections, one nocardial bronchitis, one pertussis, and one interstitial pneumonitis).

**Table 2:** Classification of non-BRC

		**Non-BRC 64 (20/44)**					
**AC**	**27 (5/22)**	**SBS**	**7 (4/3)**				
AC	17 (4/13)	SBS	5 (3/2)	GERD	12 (4/8)	Miscellaneous	6 (0/6)
AC + SBS + GERD	1 (01)	SBS + GERD	2 (1/1)			Undiagnosed	12 (7/5)
AC + GERD	9 (1/8)						

*AC*, Atopic cough; *SBS*, Sinobronchial syndrome; *GERD*, Gastroesophageal reflux disease; numbers within parentheses indicate males and females.

The RDs who had a single diagnosis at steps 1–3 were selected for basic comparison (Table 3). Although the duration of cough tended to be shorter in GERD patients, there was no significant difference between groups. Fifteen patients with BA, 26 with CVA, and 10 with NHBRC were atopic. There was no significant difference in serum IgE levels between these groups. A PND symptom was significantly more prevalent in patients with SBS than in other groups. PC_20_ was significantly lower in BA and CVA than in the other groups. There was no significant difference in %VC. The %FEV_1_/FVC ratio was lower in BA than in AC, CVA and NHBRC.

**Table 3:** Basic data

	**BA**	**CVA**	**NHBRC**	**AC**	**SBS**	**GERD**
	**(n = 19)**	**(n = 47)**	**(n = 30)**	**(n = 17)**	**(n = 5)**	**(n = 12)**
Duration (days)	421 (898)	195 (428)	491 (1194)	654 (1406)	399 (396)	97 (39)
IgE ( IU/ml)	1181 (3349)	291 (583)	262 (542)	255 (383)	127 (198)	139 (168)
PND (%)	26	32	18	29	100*	17
PC_20_ (mg/ml)	4.347 (5.820)*	4.149 (2.944)*	>20	>20	>20	>20
%VC (%)	113 (18)	106 (15)	112 (13)	118 (17)	105 (20)	115 (13)
%FEV_1_/FVC (%)	72 (11)*	81 (11)	83 (7)	83 (7)	75 (8)	82 (6)

Data are means (SD). Patients with a single diagnosis were selected for comparison. IgE, serum IgE level; PND (%), percentage of patients with postnasal drip; PC_20,_ methacholine concentration which induces a 20% decrease in the forced expiratory volume in one second (FEV_1_); %VC, percentage of predicted value of vital capacity; %FEV_1_/FVC; FEV_1_/FVC ratio expressed by percentage. * p < 0.05.

### Functional differences in BRC

**Figure 2:**
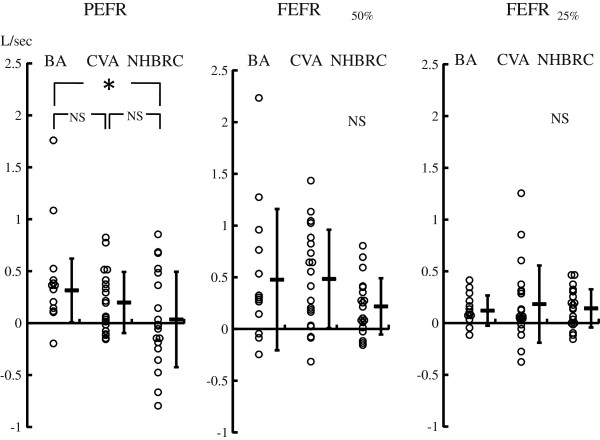
**Changes in flow volume parameters before and after β**_**2 **_**agonist inhalation.** PEFR, FEFR_50%_ and FEFR_25%_ stand for peak expiratory flow rate, forced expiratory flow rate at 50% of vital capacity and forced expiratory flow rate at 25% of vital capacity, respectively. * P < 0.05.

Among 96 RDs at step 1, 13 BAs, 10 CVAs and 19 NHBRCs received the airway reversibility test. Although baseline PEFR and FEFR_50%_ tended to be lower in CVAs, there were no significant differences between groups (Table 4). PEFR increased significantly after inhalation of salbutamol in BA and CVA patients, but not in NHBRC patients. FEFR_50%_ and FEFR_25%_ significantly increased after inhalation of salbutamol in all BRC groups. The increase of PEFR of BA patients was significantly larger than that of NHBRC (Figure 2). There was no significant difference in the increase of FEFR_50%_ and FEFR_25%_ between groups (Figure 2).

**Table 4:** Flow volume parameters

	**BA**		**CVA**		**NHBRC**	
	**Before**	**After**	**Before**	**After**	**Before**	**After**
PEFR	7.51 (2.39)	7.79 (2.37)*	6.24 (1.02)	6.48 (1.27)*	7.67 (1.87)	7.72 (1.61)^NS^
FEFR_50%_	2.85 (1.71)	3.33 (1.93)*	2.81 (0.54)	3.05 (0.80)*	3.01 (0.66)	3.24 (0.64)*
FEFR_25%_	0.80 (0.76)	0.92 (0.77)*	0.80 (0.54)	1.04 (0.80)*	0.87 (0.46)	1.03 (0.50)*

Data are means (SD). *PEFR*, peak expiratory flow rate; *FEFR*_*50%*,_ forced expiratory flow rate at 50% of vital capacity; *FEFR*_*25%,*_ forced expiratory flow rate at 25% of vital capacity. * p < 0.05, NS, not significant.

## Discussion

In the current study, we found that chronic cough diminished partially or completely in 59% of the patients who used inhaled β_2_ agonist followed by ICS. The percentage of BRC was higher than that in the previous literature [11,14], probably because we used the bronchodilator with a higher dose. CVA was the major cause of chronic cough in our study, as previously reported in Japan [15,16] although this observation is quite different from those in Western countries [17]. In the current study, on the other hand, there was a distinctly different group of patients who responded to beta agonist but did not have airway hyperresponsiveness measured by the methacholine challenge test. We named this condition NHBRC, and it consisted of a significantly large part of BRC (30%) with a higher prevalence in women (Table 1). Fujimoto et al. reported a similar phenomenon in a limited number of patients who responded to a beta agonist without airway hyperresponsiveness [18]. Although the airway reversibility test estimated by the change in FEV_1_ was not positive in all BRC patients, there was a difference in the flow volume parameters among BA, CVA and NHBRC. A small but significant increase was observed in PEFR after inhalation of salbutamol in BA and CVA, but not in NHBRC (Table 4). There was a significant difference in the magnitude of increase in PEFR between BA and NHBRC (Figure 2). These results suggest that bronchoconstriction or increased smooth muscle tone seem to occur less in NHBRC than in the other BRCs. On the other hand, FEFR_50%_ and FEFR_25%_, which are used for estimating flow limitation in the peripheral airways, were significantly increased after salbutamol inhalation in all BRC, and the magnitude of increase among BA, CVA and NHBRC was comparable. Since cough diminished after procaterol inhalation in these patients with BRC, bronchoconstriction of peripheral airways may be the common cause of chronic cough. Although bronchoconstriction does not have a direct effect on the sensitivity of the cough receptor in healthy subjects [19], bronchoconstriction or increased bronchial tone itself may stimulate the afferent tussive nerve, possibly by deformation of the airway epithelium in the peripheral airways [20]. Airway remodeling in BA and CVA has been documented [21], and similar remodeling could be involved in NHBRC inducing exaggerated airway wall deformation during bronchoconstriction so as to cause chronic cough.

Takemura et al., who compared classical BA and CVA, reported that 15% of CVA patients developed BA after two year observation and that these patients had a higher IgE level than CVA patients who did not develop BA [22]. Their results indicated that the severity of atopic status may be associated with the development of classical asthma with wheezing. Since patients with NHBRC had a significantly higher IgE level as with BA and CVA, it is possible that they could develop CVA or even BA in the future.

As shown in Table 1, there was no combination of diagnoses between BRC and AC. Since ICS was used for BRC in step 1 treatment, and ICS was also effective for AC preventing this combination. Even so, the number of AC patients in our study was far less than was reported by Fujimura et al. [11]. According to the diagnostic criteria of AC [9], patients with AC do not respond to beta agonist. The dose of beta agonist for diagnosis, however, is not defined. In the current study, twenty out of 33 NHBRC patients were atopic. If the patients with atopic NHBRC in our study were undertreated with β_2_ agonists, they could well have been diagnosed as AC. Then, the percentage of AC would have become approximately 27%, which is close to the percentage of AC in the previous study by Fujimura et al. [11]. Non-asthmatic eosinophilic bronchitis (NAEB), which shares similar clinical characteristics with AC, is one of the common causes of chronic cough [23]. NAEB is also similar to NHBRC with respect to the effectiveness of ICS and the lack of airway hyperresponsiveness. Since we did not measure the eosinophil counts, it is difficult to speculate whether eosinophilic airway inflammation was involved in NHBRC. On the other hand, the response to beta-agonist has not systematically been investigated in patients with NAEB. Therefore, it seems to be very important to investigate whether there are overlaps between NAEB, AC and NHBRC, since a part of patients with NAEB were reported to develop more serious conditions such as BA or airflow limitation during the few years of follow up [24].

Postnasal drip syndrome has been recognized as one of the major causes of chronic cough [25]. The guideline issued by the American College of Chest Physicians suggested the use of the term “Upper airway cough syndrome (UACS)” instead of PND [26]. In the current study, we did not categorize the patients as UACS since most of the cough was diagnosed otherwise by the systematic treatment cascade. Although PND was observed in 17-32% of BA, CVA, NHBRC, AC, and GERD patients (Table 3), the cough diminished without H1-antagonist in all groups except for AC. Five out of the 17 ACs had PND (Table 3) and could be diagnosed as having UACS, since both UACS and AC respond to the H1-antagonist, suggesting that there could be an overlap between UACS and AC. Moreover, it is not clear that PND itself is the independent cause of chronic cough since PND from allergic rhinitis or rhinosinusitis is frequently associated with airway disorders such as asthma [27,28], and the cough diminishes after treatment of asthma. O’Hara and Jones suggested that PND due to rhinosinusitis without a coexistent chest disease is not a predominant cause of chronic cough [29]. In the current study, all the patients with SBS had PND, as well as a productive cough with phlegm, and responded well to the treatment using clarithromycin and carbocysteine suggesting lower airway involvement. Kohno et al. classified the lower airway involvement in SBS into three categories; chronic bronchitis, bronchiectasis, and diffuse panbronchiolitis [9]. Although it is not known why there are more SBS patients in Japan compared with Western countries, the genetic factors may partially contribute to the discrepancy [30].

GERD is one of the most common causes of chronic cough and is reported to cause up to 41% of it [25]. However, Fujimura et al. reported that only 2% of chronic cough was caused by GERD in Japan [11]. In the current study, 6.5% of chronic cough was caused by GERD alone, and 10.9% of patients had GERD in combination with other disorders (Tables 1 and 2). This result shows that the percentage of GERD is altogether similar to the results in the literature from Western countries [25]. The prevalence of GERD in Japan is increasing since the end of the 1990s [31,32], possibly due to the change in dietary style and to the decrease in *Helicobacter pylori* infection brought about by antibiotics treatment. Therefore, the discrepancy between the previous study by Fujimura et al. [11] and ours could be due to the increasing occurrence of GERD, since our study period was several years later than theirs.

## Conclusion

In the current study, we started by diagnosing chronic cough with a bronchodilator. This method is inexpensive and effective for categorizing the etiology of chronic cough. The majority of patients with chronic cough had BRC. NHBRC, a new chronic cough entity, was the major cause of BRC. GERD is a common cause of chronic cough in Japan, as it is in Western countries. AC and SBS are also the causes of chronic cough in Japan.

## Abbreviations

BRC: β_2_ agonist responsive cough; HBRC: Hyperresponsive and β_2_ agonist responsive cough; NHBRC: Non-hyperresponsive and β_2_ agonist responsive cough; BA: Bronchial asthma; CVA: Cough variant asthma; GERD: Gastroesophageal reflux disease; SBS: Sinobronchial syndrome; AC: Atopic cough; PND: Postnasal drip; UACS: Upper airway cough syndrome; PPI: Proton pump inhibitor; RD: Responder; PR: Partial-responder; NR: Non-responder; ICS: Inhaled corticosteroid; ACEI: Angiotensin converting enzyme inhibitor

## Competing interests

This study was supported by the research fund of Fujita Health University, which served to cover the costs of transportation and hotel accommodations for the Conference and the financing of manuscripts, including the article processing charge. There are no competing interest with the university or any other organizations which are related to the patients who participated in this study.

## Authors’ contributions

HS designed the study and wrote the first draft under the supervision of MO. MH, YS, YM, YT, FS and HS participated in the study by recruiting the patients, and by giving suggestions for the first draft. KN participated in the study by diagnosing sinobronchitis from a specialist’s point of view as an otolaryngologist. All authors have read and approved the final manuscript.
